# Time-Efficient RSA over Large-Scale Multi-Domain EON

**DOI:** 10.3390/s24216802

**Published:** 2024-10-23

**Authors:** Tong Xi, Xuehua Li, Xin Wang

**Affiliations:** Institute of Intelligent Communication and Computing, School of Information and Communication Engineering, Beijing Information Science and Technology University, Beijing 102206, China; dongmiangka@163.com (T.X.); lixuehua@bistu.edu.cn (X.L.)

**Keywords:** time-efficient routing, branch bound, routing and spectrum allocation, multi-domain elastic optical network, multi-domain network

## Abstract

The poor timeliness of routing has always been an urgent problem in practical operator networks, especially in situations with large-scale networks and multiple network domains. In this article, a pruning idea of routing integrated with Dijkstra’s shortest path searching is utilized to accelerate the process of routing in large-scale multi-domain elastic optical networks (EONs). The layered-graph approach is adopted in the spectrum allocation stage. To this end, an efficient heuristic algorithm is proposed, called “Branch-and-Bound based Routing and Layered Graph based Spectrum Allocation algorithm (BBR-LGSA)”, which is an integrated RSA algorithm. Notably, the significant reduction in algorithm time complexity is not only reflected in the pruning method used in the routing stage but also in the construction of auxiliary graphs during the spectrum allocation stage utilizing the Branch-and-Bound method. Simulation results show that the proposed BBR-LGSA significantly reduces the average running time by nearly 78% with higher spectrum utilization in large-scale multi-domain EONs, compared with benchmark algorithms. In addition, the impact of key parameters on performance comparisons of different algorithms is evaluated.

## 1. Introduction

**Figure 1:**
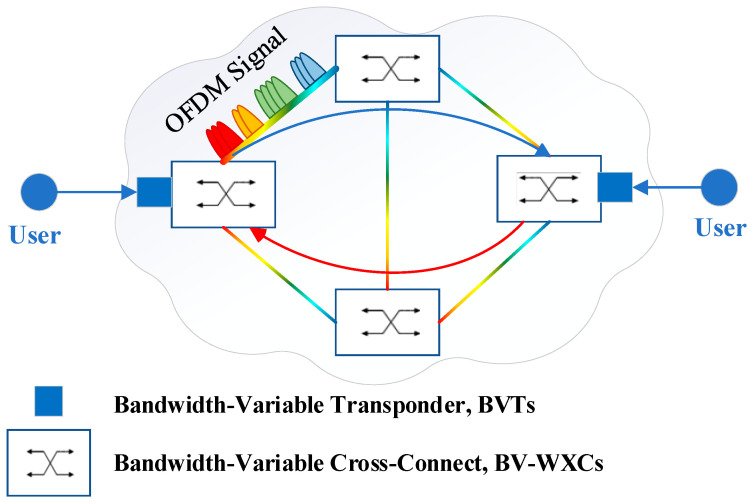
The infrastructure of an EON.

The new-generation Internet network is facing a rapid increase in traffic demands [1]. Operators are seeking more efficient and flexible technologies to meet the explosive traffic growth. However, traditional wavelength-division multiplexing (WDM) with rigid granularity makes it hard to carry the increasing traffic demands under high resource utilization. To this end, the “Elastic optical network (EON)” enabled with orthogonal frequency division multiplexing (O-OFDM) technology is considered an ideal solution for the operator network to cope with the growing traffic demands [2]. As shown in Figure 1, such EON architecture enables the accommodation of sub-wavelength, super-wavelength, and multiple rate data traffic, which presents the elastic bandwidth variation for the efficient utilization of network resources and enhanced survivability [3,4,5,6,7]. Moreover, enabling with O-OFDM technology, optical resources can be divided into finer granular frequency slots (FSs), i.e., 6.25 GHz, or even lower, in order that spectrum resources can be dynamically allocated on demand to each connection request, optimizing network efficiency and resource utilization [4,5,8].

In practical scenarios, communication nodes within the network are often geographically dispersed and may be managed by various network operators [9]. To enhance network scalability, large optical networks are commonly divided into multiple autonomous domains, where independent resource management capabilities are possessed to ensure significant autonomy and privacy. This is widely recognized as a multi-domain elastic optical network (MDEON) [10,11,12]. In MDEON, one of the most crucial problems in terms of network provisioning is routing and spectrum allocation (RSA), which constrains spectrum resource allocation: (1) Continuity: The bandwidth resources allocated to any service must be contiguous and adjacent. (2) Consistency: If the number of links used by any service is not less than two, the index value of the occupied frequency slot on each link must be the same. (3) Non-overlapping: At any time, any FS can be occupied by only one service [12,13,14]. RSA’s goal is to distribute a physical path for each demand on the spectrum, to optimize the spectrum resource utilization [14,15,16]. However, spectrum fragmentation is generated in this process, which will cause bandwidth congestion. And unreasonable spectrum allocation will decrease network resource utilization, which will affect the network performance, QoS, and a series of serious consequences. Notably, the current operator network is facing low routing computation efficiency or timeout issues, when there is a large amount of data connected to the data center or office, which further affects the user service experience. Therefore, for different service requirements, the problem of how to improve the timeliness of routing and user QoS and effectively implement time-efficient RSA has garnered significant attention from many researchers. Many optimization strategies have been proposed and validated, including RSA algorithms, and predictive models based on deep learning [17,18]. This article mainly solves the problem of poor timeliness in routing calculation over the large-scale MDEON, aiming to reduce the running time of routing calculation in large-scale MDEON.

In addition, research on RSA can be approximately classified into (a) offline RSA, whereby the traffic demands are known in advance, and physical path and contiguous spectrum slots are allocated for each demand so as to minimize the total amount of the allocated spectrum (either over the whole network or on any link), and (b) online RSA, in which a sequence of client requests arrive in some random ways [19]. In this paper, we mainly discuss the offline RSA over large-scale and multi-domain networks.

### 1.1. Related Work

In previous research, F. Pederzolli et al. [20] proposed a heuristic RSA algorithm called Minimize-Fragmentation-1 (MF1). MF1 is based on the wasted–unusable–free ratio (WUFR) to minimize a fragmentation metric by analyzing all feasible slot assignments for an incoming connection over just the shortest usable path. M. Chen et al. [21] proposed a deep-learning-based RSA strategy (D-RSA), which introduced a double deep neural network model to train RSA, and found out the link with the fewest spectrum fragmentation from the link with the least number of nodes, thereby improving network performance. Xin Wang et al. [22] studied the network-coding-based RSA algorithm (TD-RSA-CNCMU) for hybrid services in flexible optical networks. The algorithm used the layer-graph method to find the topology that met the requirement of the service’s FS requirements. However, the above research mainly optimized the spectrum utilization and did not consider the timeliness of the routing algorithm.

To solve the routing timeliness problem, Li et al. [23] studied an adaptive segmented routing (ASR) algorithm. ASR worked with a fast signaling provisioning (FSP) process that enabled lower end-to-end latency in both single-domain and multi-domain scenarios. However, this method mainly relies on reducing signaling delay; it does not reduce service path calculation time. Hsu et al. [24] proposed a heuristic algorithm named LG-SP based on the layered-graph model. The substantial performance gain was from 46% to 57% in the execution time per request in LG-SP as compared with SSR. This might originate from adequate redundant graph elimination and efficient layered-graph update. A. F. dos Santos et al. [25] proposed the Yen-BSR-SLICE (YBS) algorithm for dynamic RSA to reduce the light path blocking probability and achieve significantly improved spectrum efficiency. However, these works are not good at solving the routing problem in a time-efficient manner in large-scale networks with multiple domains.

In order to meet the challenges brought by the diversification of current actual network scenarios, Xin Wang et al. [26] proposed a multifiber-based RSA method by designing costs to select routes, spectrum slots, and first-fit fibers to accommodate requests. The simulation results showed that the proposed RSA method for multi-fiber EON was suitable for providing end-to-end optical paths through multiple domains. Y. Zhou et al. [27] proposed a new link-state-aware (LSA) RSA strategy to guarantee the Quality of Transmission (QoT) requirements under different link states by taking different layer information into account. Md Israfil Biswas et al. [28] proposed a method for Software-Defined Network (SDN) path assignment across multi-domain optical networks. The distributed system architecture was used to solve the long-standing problem of inter-domain path allocation. F. Li et al. [29] addressed an effective RSA algorithm in multi-domain heterogeneous Software-Defined Optical Networks (SDONs) under the consideration of different service QoS. Y. Li et al. [30] designed the PE-H-RSA algorithm bundle in order to achieve energy saving and QoS support. Although these heuristic algorithms consider multi-domain and QoS, they are only verified by simulation on small-scale networks (such as a 14-node NSF network) and may not be suitable for large-scale and multi-domain optical networks from real operators.

Then, H. Guo et al. [31] proposed a delay-aware RSA algorithm based on the scheduling of differentiated services with dynamic virtual topology construction (QVT-RSA) in the Software-Defined Elastic Optical Network (SD-EON). This algorithm can construct continuous dynamic virtual topology by using the parallel resource in a parallel manner for defragmentation. Therefore, it can be used for broadcast and multicast routing. QVT-RSA has the potential to be applied to MDEON, but it may not be suitable for large-scale networks. Y. Hirota et al. [12] proposed Spectrum-Constraint Path Vector Searching (SPV) to find a near-optimal RSA solution, which could attain lower blocking probability. However, since the computational complexity is exponential to the network scale, this kind of RSA algorithm is not applicable to large-scale networks. H. Xuan et al. [32] tried to improve the classical RSA algorithm by quantifying link weight, introducing quantitative values to seek the shortest path, and using first fit to allocate FSs. X. Chen et al. [33] proposed the optimized FL-SPR and FL-KSPR schemes to reduce the computational complexity in the routing step. Additionally, a TFSA scheme considering both service centralization and spectrum fragmentation was proposed in the spectrum allocation step. This scheme can make more efficient use of spectrum fragments and accommodate inbound services more efficiently. Those algorithms can effectively improve spectrum utilization and reduce bandwidth blocking probability, but they are not involving multi-domain scenarios. Li et al. [34] introduced DeepCoop, a model in which individual domains are independently managed by distinct Deep Reinforcement Learning (DRL) agents. Adjacent agents collaborated by exchanging information, thereby facilitating cooperative resource management, whereas cooperation is limited to adjacent domains.

To sum up, although the above papers studied efficient algorithms for end-to-end services in single-domain or multi-domain optical networks, few have considered the large-scale and multi-domain optical networks in the real world and studied their time-efficient routing algorithms.

### 1.2. Contributions

The optical network for the operator will evolve toward a large-scale multi-domain EON. However, most existing research rarely solves the problem of poor timeliness in routing calculation over EON (MDEON). This paper studies the time-efficient RSA with the objective of reducing the running time of routing in large-scale MDEON. The main contributions of this paper are as follows:

(a) This work presents a heuristic algorithm, BBR-LGSA, designed to manage service requests in large-scale MDEONs by optimizing both routing and spectrum allocation. The BBR-LGSA algorithm enhances routing efficiency by improving time performance and minimizing spatial complexity. By employing a Branch-and-Bound strategy, the algorithm systematically evaluates and prunes suboptimal or unnecessary routing paths, thereby reducing the search space and computational burden. This pruning mechanism is particularly crucial for large-scale, multi-domain elastic optical networks.
(b) This work adopts a layered-graph approach for efficient spectrum allocation. The layered-graph method addresses spectrum continuity and consistency issues by constructing auxiliary graphs, thereby simplifying and accelerating spectrum resource allocation under real-world network constraints.

### 1.3. Paper Organization

The rest of this article is structured as follows. Section 2 discusses the time-efficient routing and spectrum problem under the constraints of spectrum continuity and consistency in large-scale MDEON. Section 3 presents a heuristic algorithm designed to solve the problem efficiently. Section 4 presents the simulation results, with an analysis of the proposed algorithm’s performance in comparison to existing methods, as well as a discussion of key improvements, study limitations, and potential directions for future research. Section 5 concludes the whole work.

## 2. Problem Statement of Time-Efficient Routing and Spectrum in MDEON

**Figure 2:**
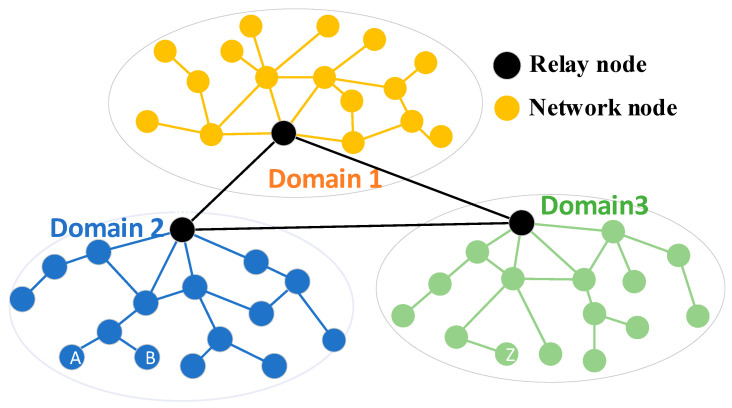
Example diagram of the MDEON.

EONs for real operators are facing great challenges due to the poor timeliness in routing and spectrum allocation processes, especially in large-scale MDEON, resulting in low-quality services and higher algorithm computational complexity. Moreover, EONs for real operators have multi-domains, and spectrum allocation should be subjected to multiple spectrum continuity constraints, which impose more challenges on the RSA processes [34]. Specifically, we should not only determine the routing connection between the source node (i.e., operator end office) and its relay node within its network domain but also establish the optimal connection between relay nodes among multiple network domains. For instance, to establish the routing connection between node A and node B, the routing processes are executed within one network domain, as shown in Figure 2. However, for nodes A and Z in different network domains, we cannot directly establish the routing connection in the same network domain. We should first find the respective relay nodes of node A and node Z. Then, the connection between the end node A and its relay node M should be established, and the optimal routing connection between the end node Z and its relay node N within its network domain should be found. Lastly, the optimal routing connection between the relay nodes M and N should be established, and the optimal routing connection between nodes A and Z across different network domains should be determined, which can be described as A to M, Z to N, and M to N.

In addition, existing RSA research mostly focuses on how to improve the spectrum utilization of RSA, with few considering both resource utilization and routing timeliness optimization simultaneously. The existing RSA strategies do not fully address the timeliness of the routing process in the network across multiple domains. It is urgent to solve the RSA problem in large-scale MDEON. In detail, RSA in large-scale MDEON comprises some sub-problems: (1) to find the optimal routing connection between the source node and its relay node within its network domain, (2) to establish the optimal path between the destination node and the relay node belonging to the destination node, (3) to establish the connections of relay nodes between the network domains, and (4) to allocate the spectrum slots considering the constraints of spectrum continuity and consistency.

## 3. Proposed BBR-LGSA Algorithm

In order to achieve the time-efficient RSA over large-scale MDEON, BBR-LGSA is presented in an efficient manner. Specifically, the Branch-and-Bound method is leveraged to accelerate the routing process. The layered-graph approach is utilized during the spectrum allocation stage. The detailed processes of the proposed BBR-LGSA algorithm are shown as follows.

### 3.1. Spectrum-Allocation-Based Layered-Graph Method

Spectrum allocation is carried out by the layered-graph method in the following steps. The first four steps elaborate the procedure of layered-graph-method-based spectrum allocation. The last two steps explain that the layered graph satisfies the accessible paths from the source node $s_{i}$ to the destination node $d_{i}$.

Step 1: Several parameters are initialized, such as the network topology of G={V,E}, and the index storage vector of I.

Step 2: For the *i*-th service requested, we find several links and nodes that satisfy the FS requirement under spectrum constraints starting from the first FS index of F. Such selected links and nodes are saved in the *m*-th auxiliary layer graph, indicated as $G^{m}=\{V^{m},E^{m}\}$, and $\forall m\in \left[1,F-n_{i}+1\right]$.

Step 3: Then, the same process of Step 1 is implemented starting from the next index in F, and $G^{m}$ is obtained.

Step 4: The above steps are repeated until all FS indexes are traversed, and the $F-n_{i}+1$ number of the auxiliary layer graph set is obtained which satisfies the spectrum request.

Step 5: According to the *m*-th auxiliary layer graph $G^{M},\forall m\in \left[1,F-n_{i}+1\right]$, we find if there are accessible paths in $G^{M}$ from source node $s_{i}$ to the destination node $d_{i}$. If it is, $G^{M}$ is saved. Otherwise, $G^{M}$ is deleted. $G^{M}$ is updated.

Step 6: Repeat step 5 until all $G^{M},\forall m\in \left[1,F-n_{i}+1\right]$ are traversed. We obtain the $G^{M}$ meeting with the required spectrum slot number and accessible paths between the source and destination node pair.

### 3.2. Routing Process Utilizing the Branch-And-Bound Method

**Figure 3:**
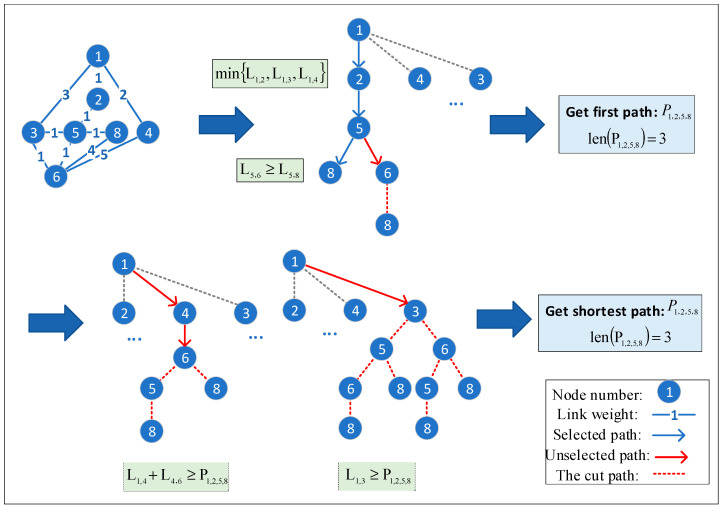
The BBR-LGSA algorithm disassembles the network topology.

In the routing stage, we use a method diagram to describe the routing process utilizing the Branch-and-Bound method. As shown in Figure 3, the branching principle effectively reduces the data amount of route search. It restricts the search process and terminates further exploration of subspaces where an optimal solution is impossible.

Specifically, in the process of expanding a node, when the path length from the source node to the current node is greater than the current shortest path length, the algorithm prunes the subtree of that node. Thus, the BBR-LGSA algorithm can consider effective data cutting at the data layer to reduce node data involved in calculations. For instance, when establishing the shortest path between source node 1 and destination node 8 is required, the path 1→2→5→8 under node 2 is found, and the path under node 3 is compared. As shown in Figure 3, we can observe that the length of path 1→3 is larger than 1→2→5→8, which means even if there is a path to destination node 8 under node 3, it is not the shortest path. Therefore, both child node 3 and the corresponding branch are cut off. Similarly, node 4 and its corresponding branches are cut off.

Similarly, when the link spectrum resources cannot meet the service requirements, or the consistency or continuity cannot guarantee non-overlap of the spectrum, these branches will not be explored.

Through the automatic scheduling of optical fiber routes in multi-domain transmission networks, the shortest path calculation function of inter-domain routes is realized. Based on the layered-graph method, an efficient heuristic RSA algorithm is proposed called “Branch-and-Bound based Routing and Layered Graph based Spectrum Allocation algorithm (BBR-LGSA)”. The real network is modeled as a directed graph G (V, E), where V is the set of nodes, and E is the set of edges. The detailed processes of BBR-LGSA are described below. The pseudo-code of BBR-LGSA is shown in Algorithm 1.
**Algorithm 1 Branch-and-Bound based Routing and Layered Graph based Spectrum Allocation algorithm (BBR-LGSA)**Input: service requests, $R_{i}=\left\{s_{i},\;d_{i},n_{i}\right\},\;\;i\in nR_{N}$, source node, $s_{i}$, destination node, $d_{i}$, the number of frequency-slots that services request, $n_{i}$;
A weighted directed graph, G = (V, E), the number of services, $\;S_{i}$
Output: Shortest path, $e_{i,st}$; Total FS resource consumption, $N_{f}$
1. **Initialize** Path costs, ${cost}_{i}$,
2. **For** each service requests $R_{i}=\left\{s_{i},d_{i},n_{i}\right\},i\in nR_{N}$,
3.  Gets the relay node A of the original domain and the relay node B of the destination domain
4.  **Initialize** graph layer sets $G^{m}=\{V^{m},E^{m}\}$, $s_{i}\in G^{m}$
5.  Build a layer graph $G^{i}$ from the Source node $s_{i}$ to the relay node A
6.  **While** A is not included in layer graph $G^{i}$ and all possible paths from $s_{i}$ to A are not found **do**
7.   **If** link e meets spectrum consistency, continuity, non-overlap, and $n_{i}$, **then**
8.    Add e to layer graph $G^{i}$
9.  **end if end while**
10. Starting the node back tracking stage
11. Randomly choose a path from $s_{i}$ to A in $G^{i}$, and save its link weight in W
12. **Initialize** mW = 0
13. **While** mW is the not the minimum value **do**
14.  Compare the physical distance between W and $W^{\prime }$, which $W^{\prime }$ equals to weight of links from A to its continuous neighbor nodes in paths in $G^{i}$
15.  **If** W is less than $W^{\prime }$, then
16.   mW=W, and the path related to $W^{\prime }$ is deleted from $G^{i}$
17. **end if end while**
18. Obtain the shortest path $e_{st}^{s,\;A}$ of $s_{i}$ and A
19. **if** $s_{i}$, $d_{i}$ are not in the same domain, **then**
20.  Repeat Step5 to Step 17 to obtain the shortest path $e_{st}^{A,\;B}$ and $e_{st}^{B,\;d}$
21. $e_{i,st}=e_{st}^{s,A}+e_{st}^{A,B}+e_{st}^{B,d}$
22. **if** $s_{i}$, $d_{i}$ are in the same domain, **then**
23.  Repeat Step 5 to Step 17 to obtain the shortest path $e_{st}^{B,\;d}$
24. $e_{i,st}=e_{st}^{s,A}+e_{st}^{B,d}$
25. **end if end for**
26. **Return** $e_{i,st}$
27. Calculate total FS resource consumption, $N_{f}$
28. **Return** $N_{f}$

**Figure 4:**
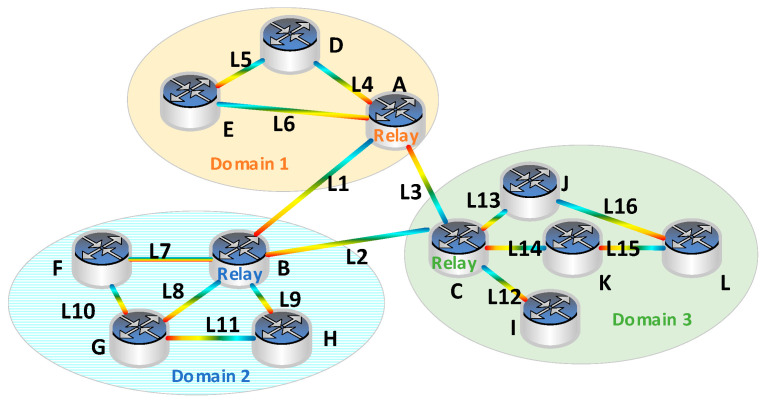
An example of a multi-domain service.

**Figure 5:**
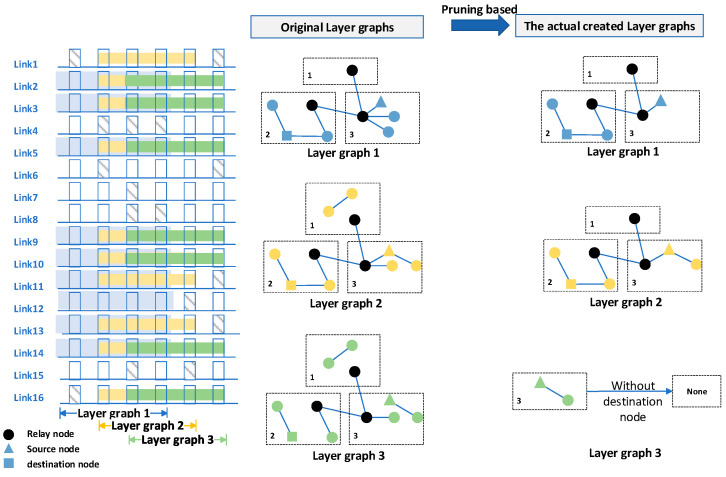
The Layered Graph Method imbued with the Pruning Concept.

An example is used to illustrate the process of the proposed BBR-LGSA. Figure 4 depicts a multi-domain optical network topology comprising 3 domains, 12 nodes named A to L, and 16 optical fiber links numbered L1 to L16. We assume that each fiber link accommodates 100 FS, and the capacity of each spectrum slot is 6.25 GHz. Meanwhile, a given service request R1 (J→G) needs four frequency slots. The shortest path algorithm determines the preliminary shortest path for R1 as F1: {L13→L2→L8}. As shown in Figure 5, as the partial occupancy of slots in link L8, service R1 cannot be established following the principles considering the constraints of spectral continuity, consistency, and non-overlapping.

**Figure 6:**
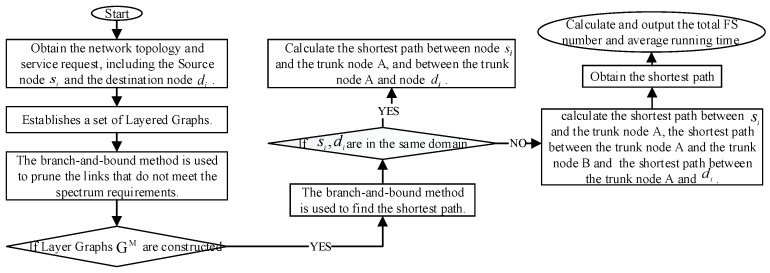
The flowchart of BBR-LGSA algorithm.

To circumvent the issue presented in the example and ensure the successful establishment of the service, the layered-graph method is considered to filter network topologies that meet the spectral requirements. Notably, by integrating the pruning concept, the connection of nodes can be directly reduced during the construction of the layered graph, thereby optimizing the solution space. Ultimately, the preliminary shortest path identified for service R1 is F1: {L13→L2→L9→L11}, which meets the spectrum requirement at this juncture. The flow chart of BBR-LGSA is shown in Figure 6.

## 4. Simulation Results and Analysis

In this section, simulations are conducted to evaluate the performance comparisons of different algorithms in terms of average running time and spectrum consumption in the test network and real operator network of large-scale MD.

### 4.1. Simulation Parameter Setup

The first-fit algorithm based on depth-first search (DFS-FirstFit), Random-Fit algorithm based on Dijkstra (Dijkstra-RandomFit), and enumeration algorithm are chosen as the benchmarks for comparisons. We mainly compare performance comparisons in terms of average running time and spectrum consumption. Simulations are conducted using Python 3.9 tools on an Intel Core i7-12700H processor, equipped with 16 GB of RAM.

### 4.2. Results of Simulation Experiments

In this section, as the parameters of service request number, domain number, network node number, and network average node degree change, we make comparisons of different approaches under different scales of MDEON scenarios.

#### 4.2.1. Performance Comparison of Different Algorithms

**Figure 7:**
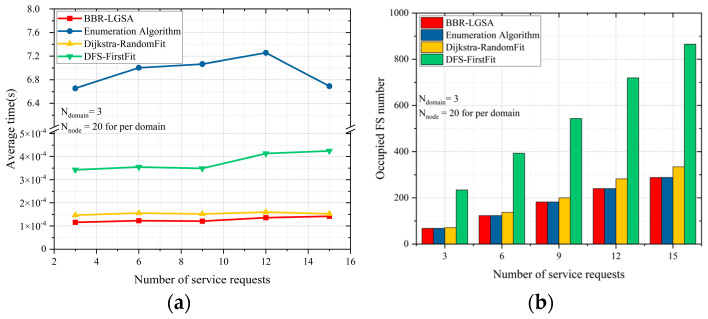
As number of service requests changes, comparison of different algorithms in terms of (**a**) average running time and (**b**) occupied FS number in small-scale networks.

In a small-scale test network, there are 3 domains and 20 nodes per domain. In Figure 7, it can be observed that our proposed BBR-LGSA algorithm reduces the average running time by nearly 16% compared to the Dijkstra-RandomFit algorithm in the small-scale network. Specifically, our BBR-LGSA algorithm consumes an average running time of 0.00013 s. The Dijkstra-RandomFit algorithm consumes 0.00016 s and fluctuates around 0.0004 s, which shows the best performance of average running time among all benchmarks. In addition, although the enumeration algorithm is the most time-consuming algorithm, it consumes the lowest number of FSs. For the spectrum consumption performance, BBR-LGSA and the enumeration algorithm occupy nearly the same amount of FS, when the number of service requests ranges from 3 to 15. However, the DFS-FirstFit algorithm outperforms other algorithms in terms of average runtime and significantly wastes FS resources compared to BBR-LGSA. It can be seen that BBR-LGSA saves about 66.32% of FS resources. This is because the DFS-Firstfit adopts the depth-first search (DFS) mechanism to search for the path that satisfies the service request. It is a blind search, and the searching time and occupied FS number are increased to some extent.

**Figure 8:**
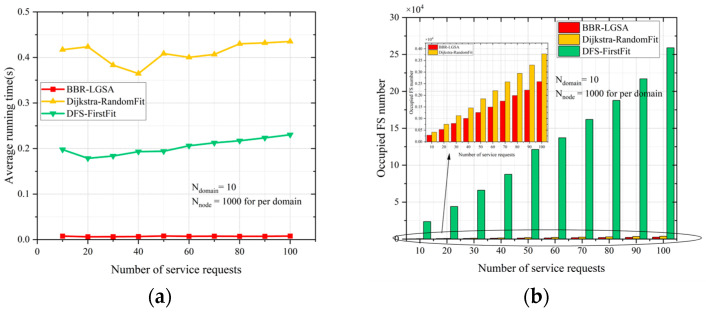
As number of service requests changes, comparison of different algorithms in terms of (**a**) average running time and (**b**) occupied FS number in large-scale networks.

The large-scale test MDEON contains 10 domains, and each domain has 1000 nodes. In Figure 8, we can see that the BBR-LGSA algorithm reduces the average running time by approximately 78.83% compared to the DFS-FirstFit algorithm in a large-scale network. Specifically, our BBR-LGSA algorithm consumes an average running time of 0.008 s, while the DFS-FirstFit algorithm consumes about 0.2 s. The Dijkstra-RandomFit algorithm consumes the most average running time. While the DFS-FirstFit algorithm can quickly find paths to meet service requests, especially when compared to other benchmark algorithms, this speed comes at the cost of significant FS resource wastage. Figure 8 shows that when handling a small number of service requests, the BBR-LGSA algorithm saves approximately 23.3% of FS resources compared to DFS-FirstFit. As the number of service requests increases, this resource saving becomes even more pronounced, with BBR-LGSA saving nearly twice the amount of FS resources. This substantial difference is due to DFS-FirstFit’s inherent inefficiency in resource allocation, where it tends to waste a large portion of FS resources for each service request.

In addition, BBR-LGSA outperforms the Dijkstra-RandomFit algorithm and the DFS-FirstFit algorithm in terms of spectrum consumption. Notably, although the enumeration method can obtain the optimal solution, it does not work in large-scale networks. Thus, the enumeration method cannot be compared with the proposed BBR-LGSA algorithm in the following large-scale test networks.

#### 4.2.2. Impact of Key Parameters on Performance Comparisons of Different Algorithms

In this section, the impact of key parameters on performance comparisons of different algorithms is analyzed. We mainly evaluate the impact of the three parameters of network node number, number of network domains, and network average node degree on algorithm comparisons.

A. Network Node Number

**Figure 9:**
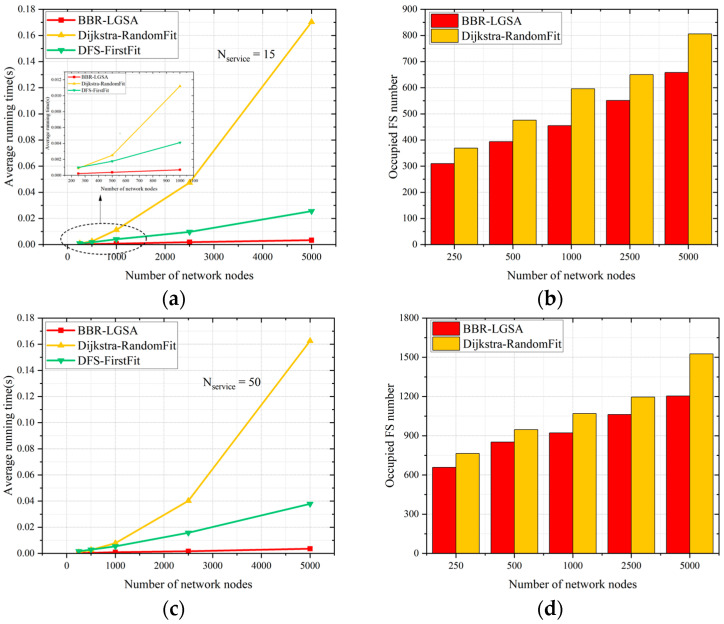
Performance comparison of algorithms based on average running time and total occupied FSs across varying network scales: (**a**) average running time for small-scale service requests, (**b**) total occupied FSs for small-scale service requests, (**c**) average running time for large-scale service requests, and (**d**) total occupied FSs for large-scale service requests.

The impact of network node number on the performance comparisons of different algorithms is evaluated under different network scales. It can be observed that the proposed algorithm outperforms the benchmark algorithms when the network node number increases in Figure 9. Specifically, the performance advantage of BBR-LGSA is more obvious in terms of average running time and spectrum consumption as the network node number increases. This is because the larger the network size, the more network segments are reduced through the pruning approach.

Comparative analysis reveals that, for both small-scale (15 services) and large-scale (50 services) service requests, the average running time by BBR-LGSA always stays around 0.0003 s. In contrast, the Dijkstra algorithm’s average running time significantly increases as the number of network nodes increases. This suggests that the Dijkstra algorithm is more suitable for smaller-scale networks. In addition, the link FS is continuously occupied by services, so it is necessary to iterate more frequently to find paths that satisfy service requests, which leads to an increase in processing time and wastage of spectral resources. The DFS-FirstFit algorithm also causes spectrum resource wastage due to the DFS search mechanism, which is not be compared with the proposed BBR-LGSA algorithm in the following large-scale test networks.

B. Network Domains

**Figure 10:**
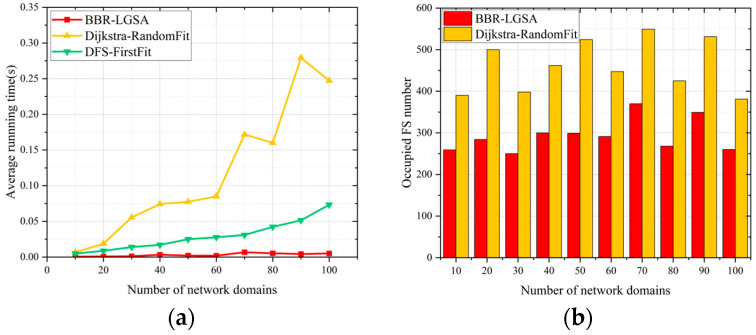
Performance comparison of different algorithms in terms of (**a**) average running time and (**b**) total number of FSs occupied as the number of domains changes.

As shown in Figure 10, the BBR-LGSA algorithm outperforms benchmarks and reduces average running time by at least 78.32% compared to benchmarks. As the number of network domains increases, the BBR-LGSA algorithm shows the best performances in the respective number of network domains.

C. Network Average Node Degree

**Figure 11:**
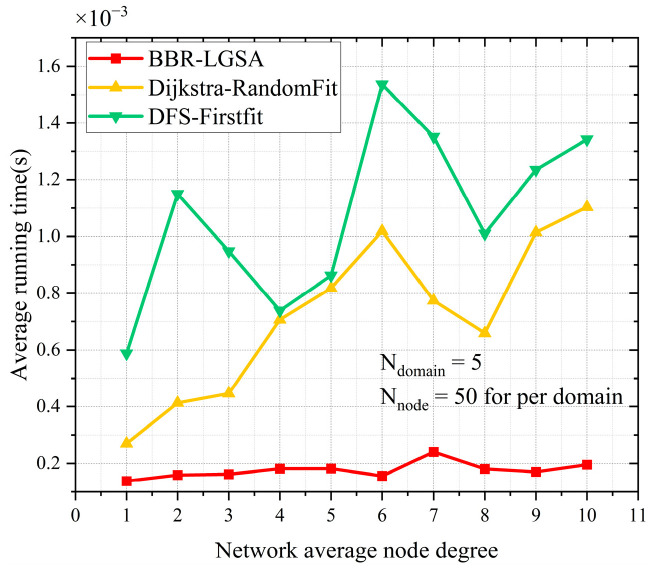
Performance comparison of different algorithms in terms of running time and total number of FSs occupied as the value of network average degree changes.

As shown in Figure 11, the BBR-LGSA algorithm maintains the best performance in terms of the network average node degree when the network average node degree increases. In addition, BBR-LGSA shows stable fluctuation in terms of average running time. However, the average running time generated by other benchmarks fluctuates significantly. Therefore, our proposed BBR-LGSA algorithm shows the best performance in terms of average running time and spectrum consumption than the benchmarks, as three different parameters increase. The changes of different parameters impact least the performance comparisons of different algorithms.

### 4.3. Real Operator Network Paradigm

Performance comparisons among different organisms in terms of routing timeliness and spectrum utilization were conducted in a real operator network. Parameters of real optical networks from operators are shown in Table 1.

To evaluate the performances of routing timeliness and spectrum efficiency of the BBR-LGSA algorithm, we consider a real operator network paradigm in China, which is a large-scale multi-domain elastic optical network.

**Figure 12:**
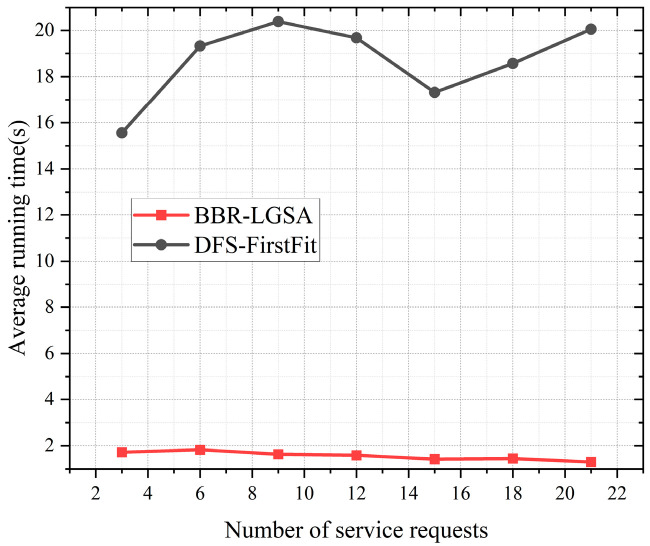
Performance comparison of different algorithms in terms of average running time and total number of FSs occupied as the number of service requests changes (real network).

As shown in Figure 12, the BBR-LGSA algorithm reduces the average running time by approximately 88.97% compared to the benchmark algorithm.

### 4.4. Discussion

This study presents the BBR-LGSA algorithm as an effective solution to the RSA problem in MDEONs. Detailed simulations showed that the algorithm significantly reduced average running time while maintaining efficient spectrum utilization compared to benchmark algorithms. Specifically, in large-scale networks, BBR-LGSA achieved up to a 78.83% reduction in running time, outperforming DFS-FirstFit and Dijkstra-RandomFit in terms of spectrum consumption. The pruning mechanism in the BBR-LGSA algorithm minimizes redundant routing calculations, making it highly scalable and time-efficient. Furthermore, the algorithm maintains stable performance across different network conditions, including variations in service request numbers, network node counts, and domain sizes.

When compared to previous research, the BBR-LGSA algorithm demonstrates clear improvements. Existing approaches, such as DFS-FirstFit and Dijkstra-RandomFit, have been widely used to address RSA problems, but they often fall short in large, complex networks. Although DFS-FirstFit can quickly find paths to meet service requests in large-scale networks, it leads to significant FS resource waste. As demonstrated by the experiment, in small-scale networks, the BBR-LGSA algorithm saves approximately 66.23% of FS resources compared to DFS-FirstFit. In large-scale networks, when handling a small number of service requests, BBR-LGSA saves approximately 23.3% of FS resources, and as the number of service requests increases, the resource savings become even more significant, nearly doubling. Dijkstra-RandomFit performs relatively well in small-scale networks but becomes computationally expensive as network size increases, especially in large MDEONs. The enumeration algorithm, while providing optimal solutions, becomes impractical in large networks due to its extensive running time. In contrast, BBR-LGSA balances both speed and resource efficiency, making it a more practical choice for real-world applications, particularly in large-scale, multi-domain networks where time and resource constraints are critical.

Despite these promising results, there are some limitations to the current study. The simulations were conducted under relatively static network conditions, which may not fully reflect the dynamic nature of real-world networks where traffic patterns fluctuate, and service demands vary over time. Additionally, while the pruning mechanism helps reduce the search space and improve performance, it may introduce computational overhead under extreme conditions, such as networks with a large number of domains or an exceptionally high volume of service requests. Another limitation is the focus on minimizing running time and spectrum consumption without considering other factors like energy efficiency, which is becoming increasingly important as optical networks continue to scale.

## 5. Conclusions

To address the time-efficient RSA in MDEON, a heuristic algorithm (BBR-LGSA) is proposed in this paper, achieving high timeliness of route processing. The Branch-and-Bound strategy aims to mitigate the spatial complexity inherent in routing search processes. This pruning approach can diminish the routing calculations. Meanwhile, the adoption of the layered-graph approach addresses the spectrum allocation challenge. Moreover, the operation effects of network node scale, domain number, network average degree, and service request number on the comparison of different methods are also evaluated by simulation. Simulation results show that BBR-LGSA outperforms all the benchmarks given in this paper, reducing average running time by nearly 78% in large-scale networks. Additionally, BBR-LGSA consistently requires less spectrum consumption. The proposed algorithm, BBR-LGSA, demonstrates significant improvements in average running time and spectrum utilization in different network densities. However, one limitation of the current work is its focus on static network conditions. In future research, we will enhance the algorithm’s adaptability to more dynamic and real-time network scenarios. Furthermore, we plan to optimize resource allocation in routing by introducing Space Division Multiplexing (SDM) or utilizing the Conventional (C-band) and Long (L-band) bands in elastic optical networks. These enhancements are expected to significantly increase bandwidth capacity and improve overall network efficiency, ensuring better performance in large-scale, multi-domain networks.

## Figures and Tables

**Table 1:** Parameters of real optical networks from operator.

Parameter Items	Node Properties	Quantity
Total number of access nodes	room	About 420,000
	installation points; wall-mounted points	About 460,000
Number of relay nodes	core convergence rooms	About 180
Number of domains	\	About 140
Average node degree	\	[2, 5]
Number of node connection relationships	\	About 400,000

## Data Availability

The data supporting the reported results are not publicly available due to privacy and ethical restrictions.
